# Health-seeking and diagnosis delay and its associated factors: a case study on COVID-19 infections in Shaanxi Province, China

**DOI:** 10.1038/s41598-021-96888-2

**Published:** 2021-08-30

**Authors:** Wenyuan Zheng, Fabrice Kämpfen, Zhiyong Huang

**Affiliations:** 1 School of Insurance, Southwestern University of Finance and Economics, Chengdu, 611130 China; 2 Population Studies Center, University of Pennsylvania, Philadelphia, PA 19104 USA; 3 Center of Health Governance and Policy, Southwestern University of Finance and Economics, Chengdu, 611130 China

**Keywords:** Health care, Risk factors

## Abstract

This time-to-event study examines social factors associated with health-seeking and diagnosis of 165 COVID-19 cases in response to the pandemic spread in Shaanxi Province, China. In particular, we investigate the differential access to healthcare in terms of delayed time from symptom onset to first medical visit and subsequently to diagnosis by factors such as sex, age, travel history, and type of healthcare utilization. We show that it takes more time for patients older than 60 (against those under 30) to seek healthcare after developing symptoms (+ 2.5 days, $p<0.01$), surveillance on people with living or travel history to Wuhan helps shorten the time to the first doctor visit (− 0.8 days) and diagnosis (− 2.2 days, $p<0.01$). A delay cut is associated with the adoption of intermediary and large hospitals rather than community-based care as primary care choices (− 1.6 days, $p<0.1$ and − 2.2 days, $p<0.05$). One unit increase of healthcare workers per 1000 people saves patients 0.5 days ($p<0.1$) for diagnosis from the first doctor visit and 0.6 days ($p<0.05$) in total. Our analysis of factors associated with the time delay for diagnosis may provide a better understanding of the health-seeking behaviors of patients and the diagnosis capacity of healthcare providers during the COVID-19 pandemic.

## Introduction

In December 2019, a series of unknown-cause pneumonia cases, later named COVID-19 by the World Health Organization (WHO), were reported in Wuhan, China, and has since spread rapidly, becoming a global pandemic within months. As of July 9th, 2021, COVID-19 has resulted in 185 291 530 confirmed cases and 4 010 834 deaths worldwide^1^.

Since the COVID-19 outbreak, there are a number of studies that look at the spread of the virus from an epidemiological perspective^2–13^, but there is minimal evidence on how individuals react to the pandemic in terms of COVID-19-related health-seeking behavior and diagnosis capacity^14^. However, like all other epidemics, the spread of COVID-19 is not only affected by its biological characteristics, such as the incubation period and transition rate but it is also influenced by social and behavioral factors^15^. Individual protective measures such as social distancing, escalation of care-seeking, and evolution of test and diagnosis protocol and capacity are all factors likely to determine the speed at which the virus spreads^16–20^. It is, therefore, crucial to understand how COVID-19-related health-seeking and diagnosis vary by socio-demographic factors to understand the progress of the pandemic better, evaluate health equity across groups, and implement effective public policies^21,22^.

In this study, we use time-to-event models to identify several key factors associated with delays of health-seeking and diagnosis in response to all COVID-19 infections in the province of Shaanxi, China, since the start of the pandemic. We measure the delay of health-seeking as the elapsed time from symptoms onset to the first doctor visit and the delay of diagnosis as the elapsed time from the first doctor visit to the diagnosis of COVID-19 infection. Key factors that we include in our analysis, such as sex, age, prefecture of residence, travel history to Wuhan, days since the first case emerged in the local community, and medical resources, are likely to be associated with both COVID-19-related health-seeking and diagnosis.

This study contributes to the existing literature in several ways. First, in addition to demographic characteristics commonly addressed in epidemiological studies^23,24^, we explore the importance of the roles played by a series of social factors in explaining the differential access to healthcare for COVID-19 patients. Second, we separately identify the likely determinants of delayed health-seeking and diagnosis, which—as our analysis suggests—are driven by different factors and should, therefore, be targeted differently.

## Method

### Data

On January 23rd, 2020, three cases, two men and one woman, 42, 22, and 32 years old, respectively, were confirmed and reported to be the first three COVID-19 cases in Shaanxi. Delays from symptom onset to the first medical visit of these cases were one, four, and one day, respectively. Delays from the first medical visit to the diagnosis of COVID-19 were two, three, and two days, respectively. Since then, the epidemic spread rapidly, reaching a maximum of 23 confirmed cases on February 5th and then contracted gradually until no new cases were detected from February 20th onward to the end of the year 2020.

**Figure 1:**
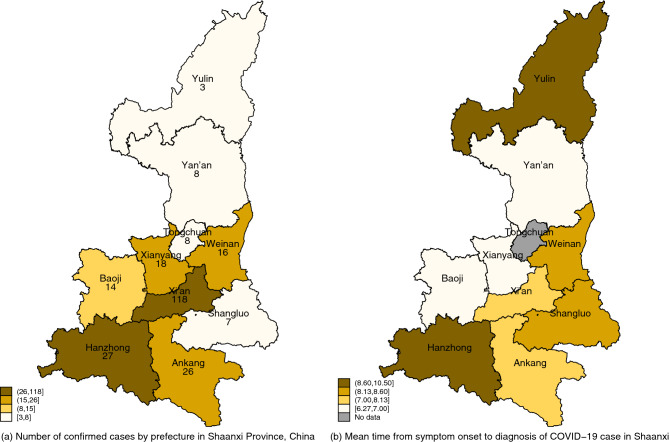
Number of COVID-19 cases and time to diagnosis in the province of Shaanxi, China. Note: Cases are collected from January 23rd to February 20th, 2020. Statistics are aggregated at the prefecture level, with one case of Hancheng included in Weinan, one case of Yangling included in Xianyang and three case of Xixian included in Xi’an.

In total, this wave of the COVID-19 pandemic between January 23rd, 2020—the date of the first confirmed case—and February 20th, 2020—the date of the last local case in 2020 resulted in 245 confirmed cases distributed in all ten prefectures with a majority of 119 cases concentrated in Xi’an, the provincial capital city, as shown in Fig. 1. A detailed account of the institutional background and epidemic progress is included in supplementary materials.

This study includes all 245 confirmed COVID-19 cases in Shaanxi Province of China till the end of year 2021. In the year 2021 up to July 11th, three new cases emerged in Shaanxi, which are excluded from this study. We extract characteristics of patients and distribution of fever clinics from published documents by the Shaanxi Health Commission. We obtain information on prefecture-level medical resources from the Shaanxi 2020 Statistic Yearbook and information on populations from the National Population Census taken in 2020.

As we are most interested in behavioral responses of patients, we exclude cases who were close contacts of other cases, therefore being isolated before developing symptoms (67 cases). We also exclude cases with incomplete information on dates of symptom onset, first medical visit, and diagnosis (9 cases). Additionally, we drop cases younger than 18 years old (4 cases). Applying these criteria generates a working sample of 165 confirmed cases.

### Variables

We focus on the elapsed time of two key events concerning health-seeking and diagnosis separately, namely delay from symptom onset to first medical visit and delay from the first medical visit to the diagnosis of COVID-19. The total elapsed time from symptom onset to diagnosis is also examined.

In addition to age and sex, we consider some risk factors including days since the first case emerged in Shaanxi, living or travel history of Wuhan, currently residing in metropolitan areas, the prefecture of residence, prefecture-level medical resources measured as the number of healthcare facilities, health workers and fever clinics *per capita* as well as the tier of the healthcare facility visited.

After announcing human-to-human transmission of COVID-19 by China CDC and reporting the first local case in Shaanxi Province, the public escalated awareness and vigilance towards the COVID-19 pandemic. Meanwhile, the government began distributing medical resources, including adopting new technology that drastically shortened the diagnosis time. Therefore, the first doctor visit and diagnosis of COVID-19 cases are likely to shorten as the pandemic progresses. We, therefore, include the number of days since the first COVID-19 confirmed case in the province, as suggested in previous studies looking at the effects of shelter-in-place policies on healthcare utilization^25^.

As COVID-19 is originated from Wuhan, people with a living or travel history to Wuhan were seen with high risk by both the public and surveillance administrators. Individuals with travel history to Wuhan may want to seek or be given medical care in priority relative to other groups of individuals. This observation motivates us to include whether individuals in our sample have traveled to Wuhan since January.

Healthcare services in China are officially classified into three tiers, with the first tier consisting of large hospitals in metropolitan areas, the second tier including intermediary hospitals located in counties and city districts, and the third tier composed of community-based facilities including clinics^26^. Medical resources are disproportionately concentrated in tier one and tier two hospitals, leaving small facilities under-resourced and understaffed. What is more, the referral system from clinic-based primary care to hospitals is not well-functioning^27^. As a result, the elapsed time from the first medical visit to the diagnosis of COVID-19 infection may depend on which type of medical facility is used by patients in the first place. Our analysis, therefore, includes the health facility tier to which the medical care used by patients belongs.

To capture unequal access to healthcare across prefectures, we also include prefecture fixed-effects in our benchmark model specification and assess the robustness of our findings by including prefecture-level medical resources such as the *per capita* number of fever clinics, health facilities, and healthcare workers in an alternative specification.

### Time-to-events models

We test a variety of parametric time-to-events models, including Weibull, exponential, Gompertz, log-logistic, log-normal, and generalized gamma distributions, and select the model with the best goodness of fit based on Bayesian Information Criterion (BIC)^28^. Using the model with the best fit, we explore risk factors associated with delayed time to medical visit and diagnosis from symptom onset and report corresponding hazard ratios and marginal effects of risk factors in median times to event.

Since cases were updated daily with the data granularity based on interval of days, the time interval between events can be interval-censored. For example, patients may see a doctor on the same day of symptom onset. In this case, the time between symptom onset and first doctor visit is interval-censored within one day. We, therefore, also fit our data using alternative parametric models that account for this kind of interval-censoring.

We report associations between risk factors and time-to-event through hazard ratios and average marginal effects of factors to the median time-to-events. We take the average across cases to obtain average marginal effects, holding other factors fixed. Data analysis is conducted using the statistical program Stata (Version 14.1, StataCorp, College Station, Texas).

## Results

### Descriptive statistics

Table 1 presents the descriptive statistics of our study sample. There were more males than females (60% vs. 40%), and age ranged between 18 and 89 with a mean value of 45.9 years old. 40% of confirmed cases had living or travel history of Wuhan. The mean time of case emergence since the first case in Shaanxi was 16.1 days. After developing symptoms, among individuals who later tested positive with COVID-19, 40% chose large hospitals (tier 1), 40% chose intermediary hospitals (tier 2), and 20% community-based services (tier 3) for the first doctor visit. Cases were geographically unevenly distributed, with up to 50% cases concentrated in Xi’an, the largest city in Shaanxi province. On average, each prefecture had 3.8 fever clinics per million inhabitants, and 10.8 healthcare workers, and 0.8 healthcare facilities per thousand inhabitants.

**Table 1:** Descriptive statistics of the study sample ($N=165$).

Variables	Mean	Median	SD	Min	Max
Time from onset to diagnosis	7.9	7	3.7	2	22
Time from onset to first doctor visit	2.3	1	2.5	0	14
Time from first doctor visit to diagnosis	5.6	4	3.6	0	22
Death	0.01	0	0.1	0	1
Female	0.4	0	0.5	0	1
Age	45.9	45	14.5	18	89
Wuhan living or travel history	0.4	0	0.5	0	1
Days since first local case	16.1	16	5.7	0	32
Large cities	0.7	1	0.5	0	1
Community based services (Tier 3)	0.2	0	0.4	0	1
Intermediary hospitals (Tier 2)	0.4	0	0.5	0	1
Large hospitals (Tier 1)	0.4	0	0.5	0	1
Xianyang	0.09	0	0.3	0	1
Shangluo	0.01	0	0.1	0	1
Ankang	0.1	0	0.3	0	1
Baoji	0.08	0	0.3	0	1
Yan’an	0.02	0	0.1	0	1
Yulin	0.01	0	0.1	0	1
Hanzhong	0.1	0	0.3	0	1
Weinan	0.06	0	0.2	0	1
Xi’an	0.5	1	0.5	0	1
Fever clinics per million	3.8	3.1	1.0	2.8	7.0
Health workers per thousand	10.8	10.5	0.9	9.8	13.1
Healthcare facilities per thousand	0.8	0.5	0.3	0.5	1.4

$N=165$. *Large cities* is a dichotomous variable that takes the value 1 if an individual lives in a city and 0 if living in counties or rural area. The prefecture of Tongchuan has no confirmed cases in this working sample.

**Figure 2:**
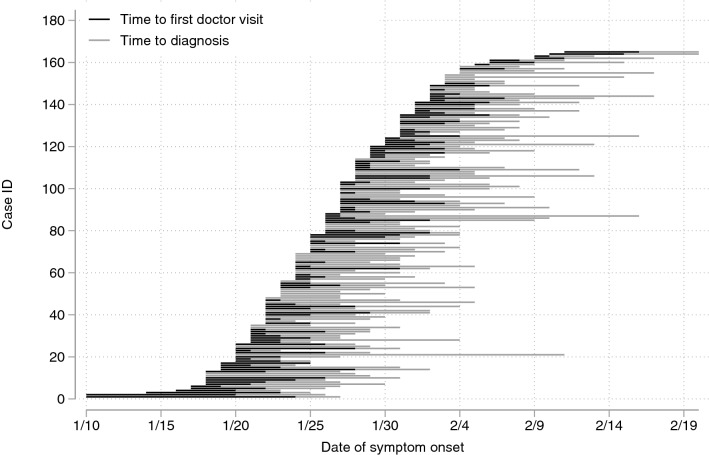
Time from COVID-19 symptom onset to first doctor visit and confirmation of COVID-19 patients.

The mean time from symptom onset to diagnosis was 7.9 days, with a maximum of 22 days. It took on average 2.3 days to seek healthcare after symptom onset and another 5.6 days to reach the diagnosis. The delay in time from symptoms onset to first doctor visit and subsequently to the diagnosis of COVID-19 infection was evolving as the epidemic progressed, as shown in Fig. 2. It took on average 11.3 for the first ten cases to be diagnosed after symptom onset compared with 6.5 days for the last ten cases. Moreover, the time delay displayed some geographical differences across prefectures, as shown in Fig. 1.

### Temporal change and factors associated with diagnosis delay

We have considered several time-to-event parametric specifications. Table 2 reports the BIC values for various parametric models we estimated, out of which the Weibull model seemed to be the one best fit our data. The results we present in this section are therefore derived using this specification.

**Table 2:** BICs of parametric models with time from symptom onset to diagnosis of COVID-19 infection.

	Weibull	Exponential	Gompertz	Log-logistic	Log-normal	Generalized gamma
BIC	296.48	468.31	329.00	305.66	305.67	301.00

The sample size $N=165$. We use a Bayesian Information Criterion (BIC) to select the parametric specification that best fits the data. Based on BIC, the model that best fits our data is the Weibull specification. We hence use that specification to estimate our statistical models.

Table 3 presents estimates of our Weibull time-to-event models in which we regress delayed time from COVID-19 symptom onset to first doctor visit and diagnosis on risk factors. The time delay to the first doctor visit shows a sharp age gradient. Older adults are likely to take more time before health-seeking compared with younger groups. It takes 2.5 more days ($p<0.01$) for those aged 60 and above to make the first doctor visit than those younger than 30 years old. However, this group of patients seems to benefit from a shorter delay on diagnosis (1.6 days shorter, $p<0.1$). The total time delay from onset to diagnosis is 0.9 days more for this group though the effect is not statistically significant.

Disease surveillance on people living in Wuhan or having a travel history to Wuhan results in 0.8 days ($p<0.1$) decrease in time to the first doctor visit and another 2.2 days ($p<0.01$) decrease in time to the diagnosis. The time elapsed since the first case, which may capture the evolution of risk perception, test capacity, diagnosis protocol, and learning-by-doing knowledge, shortens doctor visit and diagnosis time by 0.2 ($p<0.01$) and 0.1 ($p<0.05$) days per an additional day, respectively. Compared to community-based services, using intermediate and large hospitals as first doctor visits is associated with 1.6 ($p<0.1$) and 2.2 days ($p<0.05$) saving in time from the first doctor visit to the diagnosis of COVID-19 infection.

**Table 3:** Factors associated with time from symptom onset to first doctor visit and diagnosis of COVID-19 infection (with age groups).

	Time from symptom onset to first doctor visit		Time from first doctor visit to diagnosis of COVID-19		Time from symptom onset to diagnosis of COVID-19	
	Hazard ratio	Marginal effect	Hazard ratio	Marginal effect	Hazard ratio	Marginal effect
Female	− 0.1	0.2	0.09	− 0.2	0.03	− 0.09
	(0.2)	(0.4)	(0.2)	(0.5)	(0.2)	(0.5)
30–39	− 1.2***	1.3***	0.9***	− 2.5***	0.4	− 1.0
	(0.5)	(0.5)	(0.3)	(0.9)	(0.3)	(0.8)
40–49	− 1.3***	1.4***	0.4	− 1.3	0.1	− 0.3
	(0.4)	(0.4)	(0.3)	(0.9)	(0.3)	(0.8)
50–59	− 1.7***	2.1***	0.4	− 1.2	− 0.4	1.3
	(0.5)	(0.5)	(0.3)	(1.0)	(0.3)	(0.9)
60+	− 1.8***	2.5***	0.5*	− 1.6*	− 0.3	0.9
	(0.5)	(0.6)	(0.3)	(0.9)	(0.3)	(0.8)
Wuhan living or travel history	0.5*	− 0.8*	0.8***	− 2.2***	1.1***	− 3.0***
	(0.3)	(0.5)	(0.2)	(0.5)	(0.2)	(0.6)
Days since first local case	0.09***	− 0.2***	0.04**	− 0.1**	0.08***	− 0.2***
	(0.02)	(0.03)	(0.02)	(0.04)	(0.01)	(0.04)
Large cities	− 0.02	0.02	0.4	− 1.0	0.3	− 0.8
	(0.3)	(0.5)	(0.3)	(0.8)	(0.3)	(0.8)
Intermediary hospitals (Tier 2)	0.07	− 0.1	0.5*	− 1.6*	0.4	− 1.1
	(0.4)	(0.5)	(0.3)	(0.9)	(0.3)	(0.8)
Large hospitals (Tier 1)	− 0.3	0.4	0.8**	− 2.2**	0.4	− 1.3
	(0.4)	(0.6)	(0.3)	(1.0)	(0.3)	(0.9)
Xianyang	− 0.6	1.0	1.1***	− 2.8***	0.8**	− 2.2***
	(0.4)	(0.9)	(0.3)	(0.8)	(0.3)	(0.8)
Ankang	− 0.6	1.2	1.1***	− 2.6***	0.5	− 1.4
	(0.4)	(0.9)	(0.4)	(0.8)	(0.3)	(0.9)
Baoji	0.6	− 0.8	1.0***	− 2.6***	1.1***	− 2.9***
	(0.4)	(0.5)	(0.4)	(0.8)	(0.4)	(0.8)
Yan’an	− 0.3	0.5	0.2	− 0.6	0.1	− 0.4
	(0.8)	(1.4)	(0.6)	(1.8)	(0.6)	(1.8)
Yulin	− 0.09	0.1	0.4	− 1.1	− 0.04	0.1
	(0.8)	(1.3)	(0.8)	(2.1)	(0.8)	(2.5)
Hanzhong	0.4	− 0.5	0.3	− 0.8	0.2	− 0.6
	(0.5)	(0.6)	(0.3)	(0.9)	(0.3)	(0.9)
Weinan	− 0.6	1.1	0.5	− 1.4	0.07	− 0.2
	(0.5)	(1.0)	(0.4)	(1.0)	(0.4)	(1.2)
Shangluo			0.9	− 2.2	1.3	− 3.2**
			(0.8)	(1.7)	(0.8)	(1.6)
Constant	− 2.5***		− 6.3***		− 8.5***	
	(0.8)		(0.7)		(0.8)	
Mean time	2.3 days		5.6 days		7.9 days	

*$p<0.1$, **$p<0.05$, ***$p<0.01$. Results are estimated using a Weibull parametric specification and a sample of $N=165$. *Large cities* is a dichotomous variable that takes the value 1 if an individual lives in a city and 0 otherwise (living in counties or rural area). Tongchuan has no confirmed cases in this working sample. Marginal effects of risk factors to median time-to-events are calculated.

It is noticeable that prefectures such as Xianyang, Shangluo, and Baoji reported shorter times for doctor visits and diagnoses than Xi’an, the province capital city. As shown in Table 4, there exist substantial disparities in medical resources across prefectures. In particular, Xianyang and Baoji have the second and third largest health workforce *per capita* in the province. At the same time, Shangluo tops the ranking list on the number of healthcare facilities *per capita*.

To test whether this geographic difference in medical resource contributes to the inequality in delayed time for the doctor visit and diagnosis, we include a set of variables that measure medical resources of each prefecture, including *per capita* number of fever clinics, number of healthcare workers, and number of healthcare facilities. As shown in Table 5, the influence of age, travel history to Wuhan, number of days since the first cases, and types of healthcare facilities are essentially unchanged after including these additional controls in our analysis. In addition, we found that one unit increase of healthcare workers per 1,000 people saves patients 0.5 days ($p<0.1$) for diagnosis from the first doctor visit and 0.6 days ($p<0.05$) in total.

**Table 4:** Geographic distribution of medical resources.

Prefecture	Number of fever clinics	Number of healthcare workers	Number of healthcare facilities	Fever clinics per million	Healthcare workers per thousand	Healthcare facilities per thousand
Ankang	13	24,368	2927	5.21	9.77	1.17
Baoji	15	402,23	3085	4.52	12.11	0.93
Hanzhong	14	33,536	3691	4.36	10.44	1.15
Shangluo	8	211,56	2782	3.92	10.36	1.36
Tongchuan	6	11,852	871	8.59	16.97	1.25
Weinan	13	52,033	4251	2.77	11.10	0.91
Xi’an	40	136,333	7011	3.09	10.53	0.54
Xianyang	22	55,014	4329	5.22	13.06	1.03
Yan’an	16	24,298	2631	7.01	10.64	1.15
Yulin	16	36,743	3826	4.41	10.14	1.06

Statistics of fever clinics are extracted from published documents by Shaanxi Health Commission and statistics of healthcare workers and healthcare facilities are obtained from the Shaanxi Statistic Yearbook in 2020.

**Table 5:** Factors associated with time from symptom onset to first doctor visit and diagnosis of COVID-19 (with control variables of prefecture medical resource).

	Time from symptom onset to first doctor visit		Time from first doctor visit to diagnosis of COVID-19		Time from symptom onset to diagnosis of COVID-19	
	Hazard ratio	Marginal effect	Hazard ratio	Marginal effect	Hazard ratio	Marginal effect
Female	− 0.1	0.2	0.2	− 0.4	0.05	− 0.1
	(0.2)	(0.4)	(0.2)	(0.5)	(0.2)	(0.5)
30–39	− 1.4***	1.6***	0.7**	− 2.0**	0.2	− 0.6
	(0.4)	(0.5)	(0.3)	(0.9)	(0.3)	(0.8)
40–49	− 1.1***	1.3***	0.3	− 1.0	0.05	− 0.1
	(0.4)	(0.4)	(0.3)	(0.9)	(0.3)	(0.7)
50–59	− 1.6***	2.0***	0.4	− 1.0	− 0.4	1.3
	(0.5)	(0.6)	(0.3)	(0.9)	(0.3)	(0.9)
60+	− 1.8***	2.5***	0.3	− 1.0	− 0.4	1.2
	(0.4)	(0.6)	(0.3)	(0.8)	(0.3)	(0.8)
Wuhan living or travel history	0.4	− 0.6	0.9***	− 2.3***	1.0***	− 2.9***
	(0.3)	(0.5)	(0.2)	(0.5)	(0.2)	(0.6)
Days since first local case	0.08***	− 0.1***	0.04**	− 0.1**	0.08***	− 0.2***
	(0.02)	(0.03)	(0.02)	(0.05)	(0.01)	(0.04)
Large cities	− 0.2	0.3	0.1	− 0.3	0.2	− 0.5
	(0.3)	(0.6)	(0.3)	(0.7)	(0.3)	(0.8)
Intermediary hospitals (Tier 2)	− 0.3	0.5	0.6**	− 1.7**	0.3	− 1.0
	(0.3)	(0.5)	(0.3)	(0.9)	(0.3)	(0.8)
Large hospitals (Tier 1)	− 0.3	0.4	0.7**	− 2.0**	0.4	− 1.2
	(0.4)	(0.6)	(0.3)	(1.0)	(0.3)	(0.9)
Fever clinics per million	− 0.1	0.2	0.1	− 0.3	0.1	− 0.3
	(0.2)	(0.3)	(0.1)	(0.4)	(0.1)	(0.4)
Health workers per thousand	0.09	− 0.1	0.2*	− 0.5*	0.2**	− 0.6**
	(0.1)	(0.2)	(0.1)	(0.3)	(0.10)	(0.3)
Healthcare facilities per thousand	− 0.03	0.05	0.5	− 1.3	0.3	− 0.7
	(1.0)	(1.7)	(0.6)	(1.7)	(0.7)	(1.9)
Constant	− 2.5*		− 8.2***		− 10.7***	
	(1.3)		(1.3)		(1.3)	
Mean time	2.3 days		5.6 days		7.9 days	

*$p<0.1$, **$p<0.05$, ***$p<0.01$. Our results are derived using a Weibull parametric specification and a sample of $N=165$. *Large cities* is a dichotomous variable that takes the value 1 if an individual lives in a city and 0 otherwise (live in counties or rural area). Tongchuan has no confirmed cases in this working sample. Marginal effects of risk factors to median time-to-events are calculated. *Number of fever clinics*, *number of healthcare workers*, *number of healthcare facilities* are in unit per million, per thousand and per thousand covered population/inhabitants, respectively.

The alternative interval-censored data model which we use to account for the fact that symptom onset and doctor visits can be reported on the same day produces very similar results, as evidenced in Table 6.

**Table 6:** Factors associated with time from symptom onset to first doctor visit and diagnosis using interval-censored data model.

	Time from symptom onset to first doctor visit		Time from first doctor visit to diagnosis of COVID-19		Time from symptom onset to diagnosis of COVID-19	
	Hazard ratio	Marginal effect	Hazard ratio	Marginal effect	Hazard ratio	Marginal effect
Female	− 0.1	0.2	0.1	− 0.3	0.04	− 0.1
	(0.2)	(0.3)	(0.2)	(0.5)	(0.2)	(0.5)
30–39	− 1.0***	1.2***	0.9***	− 2.5***	0.4	− 1.0
	(0.3)	(0.4)	(0.3)	(0.9)	(0.3)	(0.8)
40–49	− 0.8***	0.9***	0.4	− 1.3	0.1	− 0.3
	(0.3)	(0.3)	(0.3)	(0.9)	(0.3)	(0.8)
50–59	− 1.5***	2.3***	0.4	− 1.2	− 0.5	1.4
	(0.4)	(0.6)	(0.3)	(1.0)	(0.3)	(0.9)
60+	− 1.5***	2.1***	0.5*	− 1.5*	− 0.3	0.9
	(0.3)	(0.5)	(0.3)	(0.9)	(0.3)	(0.8)
Wuhan living or travel history	0.3	− 0.5	0.9***	− 2.3***	1.1***	− 3.0***
	(0.2)	(0.4)	(0.2)	(0.5)	(0.2)	(0.6)
Days since first local case	0.06***	− 0.1***	0.04***	− 0.1***	0.08***	− 0.2***
	(0.02)	(0.03)	(0.02)	(0.04)	(0.01)	(0.04)
Large cities	− 0.03	0.06	0.4	− 1.0	0.3	− 0.8
	(0.3)	(0.5)	(0.3)	(0.8)	(0.3)	(0.8)
Intermediary hospitals (Tier 2)	− 0.2	0.3	0.5**	− 1.6*	0.4	− 1.1
	(0.3)	(0.4)	(0.3)	(0.9)	(0.3)	(0.8)
Large hospitals (Tier 1)	− 0.6**	1.0**	0.8**	− 2.2**	0.5	− 1.3
	(0.3)	(0.5)	(0.3)	(1.0)	(0.3)	(0.9)
Xianyang	− 0.3	0.6	1.1***	− 2.8***	0.9**	− 2.3***
	(0.3)	(0.6)	(0.3)	(0.8)	(0.3)	(0.8)
Shangluo	5.3	− 1.9***	0.9	− 2.4	1.3*	− 3.3**
	(.)	(0.2)	(0.8)	(1.7)	(0.8)	(1.6)
Ankang	− 0.9**	1.9*	1.1***	− 2.7***	0.5	− 1.5
	(0.4)	(1.0)	(0.4)	(0.9)	(0.3)	(0.9)
Baoji	0.2	− 0.2	1.1***	− 2.7***	1.2***	− 3.0***
	(0.4)	(0.5)	(0.4)	(0.8)	(0.4)	(0.8)
Yan’an	− 0.3	0.6	0.2	− 0.7	0.2	− 0.5
	(0.6)	(1.2)	(0.6)	(1.8)	(0.6)	(1.8)
Yulin	− 0.8	1.8	0.4	− 1.2	− 0.02	0.06
	(0.8)	(2.2)	(0.8)	(2.1)	(0.8)	(2.4)
Hanzhong	− 0.2	0.3	0.3	− 0.8	0.2	− 0.6
	(0.4)	(0.6)	(0.3)	(0.9)	(0.3)	(1.0)
Weinan	− 0.5	0.9	0.5	− 1.4	0.08	− 0.2
	(0.4)	(0.9)	(0.4)	(1.0)	(0.4)	(1.2)
Constant	− 0.7		− 6.9***		− 9.1***	
	(0.6)		(0.7)		(0.8)	
Mean time	2.3 days		5.6 days		7.9 days	

*$p<0.1$, **$p<0.05$, ***$p<0.01$. Our results are derived using a Weibull parametric specification and a sample of $N=165$. *Large cities* is a dichotomous variable that takes the value one if an individual lives in a city and 0 otherwise (living in counties or rural areas). Tongchuan has no confirmed cases in this working sample. The marginal effects of risk factors on median time-to-event are calculated. These results are derived using an interval-censored data model to account for the fact that some individuals might have developed COVID-19 symptoms and visited a doctor on the same day.

## Discussion

To better control the spread of COVID-19 and limit the negative health consequences of an infection, it is crucial to avoid time delay in diagnosing an infection. Evidence shows that delayed access to health care is associated with adverse health outcomes^29^, and this is particularly true when it comes to infectious diseases such as COVID-19, for which it has been shown that delayed care is associated with heightened mortality in COVID-19 patients^30^.

Many studies have identified epidemiological factors that are associated with the spread of COVID-19^2–13,23,24^, but there is still limited evidence of the importance of social factors that explain health seeking behaviors and early diagnosis in the face of a COVID-19 infection. The main contribution of the present study is to provide evidence of the importance of social factors to explain COVID-19 health seeking behaviors and diagnosis. More specifically, our findings highlight the importance of patient age, travel history, hospital characteristics and local healthcare resources.

Our results show that older adults in Shaanxi tend to delay their first doctor visit after symptom onset. This is consistent with a study based on 14,618 Belgian COVID-19 admissions which found that people under 20 have the shortest time delay for hospitalization after symptom onset^31^. Our findings are also consistent with previous studies showing that medical care delays exist disproportionately among older adults^32–35^. Several explanations can explain the age gradient in healthcare delay. One possible explanation can be due to economic and functional limitations^36,37^.Biological and pathological factors may also contribute to the delay of treatment among older adults. Symptoms like fever are more likely to be absent for older adults with infection, and this may result in diagnostic delays^38,39^. Comorbidities, which are common for older adults^40,41^, may also complicate clinical symptoms of infection, leading to delays in seeking treatment and diagnosis^42,43^. Other possible explanations include loss of social connection and interaction^44,45^, ageism^46^ and tendency of self-medication^47,48^.

Moreover, we also find, perhaps not surprisingly, a negative association between healthcare delay and travel history to Wuhan. Disease surveillance is critical for timely interventions in public health practice^49^ and WHO urged public authorities to rapidly identify and take care for cases of COVID-19, trace and quarantine their contacts and monitor disease trends over time^50^. A study based on COVID-19 cases in Singapore^51^ confirmed that enhanced surveillance and contact tracing help reduce transmission. On the other hand, close surveillance can raise public concerns because of the possible stigmatization, discrimination^52^ and privacy issues^53,54^it can entail. The current crisis uncovered the weakness of disease surveillance in almost all countries characterized by weak public communication^53^, poor contact tracing^55^ and incomplete and inaccurate data^56^. The question as to how to build a surveillance system that is robust, efficient, but also able to safeguard individuals from stigmatization, privacy breach, and information misuse remains open however^57^.

Longer delay to COVID-19 diagnosis when patients choose community-based service as initial choice of primary care as opposed to larger hospitals may hint at the inefficiency of community services in coping with COVID-19 in Shaanxi. Though community-based healthcare and primary care at large is supposed to play a crucial role to ensure an effective COVID-19 response^58^, community and primary care systems functioned ineffectively in many countries during the height of the pandemic, often because of lack of testing and diagnosis capacities^59^. In addition to insufficient capacities, China’s primary care system faces many other challenges such as undertrained and understaffed workforce, the fragmentation of clinical care and public health service, and the disintegration between primary care institutions and hospitals^60^. Lack of diagnostic and testing capacities can potentially hurt the efficiency of primary care services^61^ and lead to other adverse health outcomes^62,63^. However, since in China patients are allowed to self-refer to hospitals without paying much more and patients often have a preference for hospitals over primary care institutions, those who do choose community-based healthcare and primary care in the first place are often from lower socioeconomic background, which can be another factor that explains healthcare delay^64^. Moreover, although the importance of health workforce on health outcomes is well-documented, there is scant evidence on the association between health workforce and diagnosis time^65,66^. Our findings therefore present some initial and suggestive evidence on the importance of healthcare resources, particularly in terms of healthcare workforce, on the timely diagnosis of COVID-19.

### Limitations

We acknowledge several limitations of our study: First, our study takes place in a country with relatively low infection and transmission risks and our results are derived from a small sample size. Our results may therefore not be generalizable to places where risks are higher and strains to health care systems more important.

Second, mild symptoms and asymptomatic cases may be unaware of their conditions and may not seek healthcare. To the extent that only individuals with relatively severe COVID-19 symptoms decided to visit a medical doctor, our analysis is likely to be biased downwards because individuals with only mild symptoms are plausibly more likely to delay doctor visits.

Third, we fail to incorporate in our analysis several important social factors such as education and income due to data availability, though we include the prefecture of residence and whether individuals live in metropolitan areas, which could serve as a proxy for socioeconomic characteristics.

Fourth, our analysis on the geographic variation in time delays for COVID-19 health-seeking behaviors would have benefited from a more formal multilevel analysis. However, given our limited sample size, such analysis could not be performed, and we are limited to consider geographic characteristics as simple fixed effects in multivariate regressions.

Finally, policy recommendations based on our findings may not apply to other contexts and countries. Both the success of disease surveillance and the failure of primary care in dealing with the current COVID-19 crisis is embedded in a more extensive system of social arrangement that is a specific and unique feature of China.

### Conclusion

This study presents some suggestive evidence on risk factors associated with a time delay from the onset of symptom to the diagnosis of COVID-19 infection. Based on a relatively small sample in Shaanxi, China ($N=165$), we have identified factors like age, surveillance on travel history, types of healthcare facilities visited, size of the health workforce, which are likely to affect either health-seeking or diagnosis or both, contributing to the time delay of proper diagnosis.

Our findings may offer some valuable insights for policymakers. Older adults tend to delay their care, emphasizing the need for closer surveillance and targeted public policies for older adults and vulnerable groups in general. The positive association between the surveillance of high-risk individuals with travel history to Wuhan and a shorter diagnosis delay supports the prioritization of surveillance on groups with higher risks of exposure. The delay caused by the utilization of community-based healthcare has two direct policy implications: First, primary care can only benefit patients if it is empowered and integrated into the extensive healthcare system. Second, policies steering patients towards primary care have to be accompanied by reforms aimed at reinforcing the primary care system.

Our analysis on the factors associated with time delays for COVID-19 diagnosis sheds light on the importance of patients’ health-seeking behaviors and diagnosis capacity of healthcare providers during the pandemic. That being said, the small sample size undermines the external validity of our study and prevents us from a thorough and more conclusive validation of the mechanisms involved. More research is needed to better understand the mechanisms—be they biological, economic, social or a combination thereof– of the age gradient in COVID-19 health seeking behaviors we observe in our data. Moreover, the “social” cost of surveillance should be weighted along with its benefit: the optimal level of surveillance should be based on scientific and factual considerations rather than political preferences and inclinations. Finally, the primary care system should be enhanced in the long run in China. Future research can guide policy makers in striking the right balance between strengthening primary care system and structure and developing modern hospitals and advanced technologies that can help tackling future public health challenges.

## Supplementary Information

Supplementary Information.
