# Supplementary material for: Health-seeking and diagnosis delay and its associated factors: a case study on COVID-19 infections in Shaanxi Province, China
Source: Sci Rep. 2021 Aug 30;11:17331. doi: 10.1038/s41598-021-96888-2 (PMC8405662; doi:10.1038/s41598-021-96888-2)
Supplement: Supplementary file 1 — Supplementary Information. [file 41598_2021_96888_MOESM1_ESM.pdf]

Health-seeking and diagnosis delay and its associated factors – a case study on COVID-19 infections in Shaanxi Province, China

Wenyuan Zheng[1], **Fabrice Kämpfen**[2], **and Zhiyong Huang**[3,*]

[1]Southwestern University of Finance and Economics, School of Insurance, Chengdu, 611130, China
[2]University of Pennsylvania, Population Studies Center, Philadelphia, PA 19104, USA
[3]Southwestern University of Finance and Economics, Center of Health Governance and Policy, Chengdu, 611130, China
[*]Corresponding author: zhiyonghuang@swufe.edu.cn

Supplementary files

S1: Institutional background and epidemic progress

As part of Northwest China, Shaanxi is a landlocked province consisting of ten prefectures, namely Tongchuan, Baoji, Xianyang, Weinan, Yan'an, Hanzhong, Yulin, Ankang, Shangluo, and Xi'an. According to the National Population Census taken in 2020, Shaanxi has 39.53 million with 51.17% men and 48.83% women, and 17.33% younger than 15 years old, 63.46% between 15 and 59 years old and 19.20% older or equal to 60 years old. Xi'an, as the provincial capital city, hosts 32.77% of the whole Shaanxi population. Shaanxi has relatively rich medical resources in terms of per-capita healthcare workers and facilities, which are above but still very close to the national average, as displayed in Figure S1.

After the outbreak in Wuhan, a series of aggressive measures have been adopted by governments of various levels under the principle of "early identification, early report, early isolation, and early treatment"[1], first in Wuhan and rapidly elsewhere in China. New hospitals dedicated explicitly to treating COVID-19 patients were built quickly in Wuhan. Thousands of doctors and nurses were deployed to Hubei province, where non-urgent medical care has been postponed in most hospitals. Transportation within Hubei was utterly shut down and widely suppressed in places out of Hubei. People were asked to stay home as much as possible and wear face masks when going out of the home. Most shops, restaurants, schools, and many public places were closed, and large gatherings were prohibited[2].

Among all measures taken, some may particularly affect individual health-seeking behaviors related to COVID-19. First to note is the restriction of COVID-19 diagnosis to special fever clinics, which were established after SARS by many hospitals and believed to be able to speed up screening for infectious diseases for patients with fevers and reduce hazards of cross-infection as the fever clinics were isolated from other wards[3]. Most of these fever clinics were maintained up to this pandemic, and new ones were set up soon after the COVID-19 outbreak as gatekeepers of early diagnosis of COVID-19 infections. On January 26th, three days after the first confirmed cases in Shaanxi, the Health Commission of Shaanxi Province officially published the list of 164 fever clinics that were available to its population and people with travel history to Wuhan or those who have been in close contact with confirmed cases or having symptoms of fatigue, fever, or cough were encouraged to seek a diagnosis in nearby fever clinics. Treatments for COVID-19 from institutions that were not on the list, as well as self-treatment by purchasing medications from pharmacies, became illegal.

In addition, extensive community-based control measures have been taken, such as regular temperature checks at the entrance of each housing community, self-report of COVID-19-related symptoms, and close monitoring of people from outside the community[4]. Finally, the Chinese central government made it clear that testing *and treatment* for COVID-19 would be free at a very early stage. The Nation Healthcare Security Administration and Ministry of Finance announced that all related charges for COVID-19 confirmed infections would be covered through public subsidy on January 22nd and then expanded these policies to suspected cases on January 27th[5].

S2: Geographic distribution of healthcare resources

Figure S1. National geographic distribution of medical resources in China

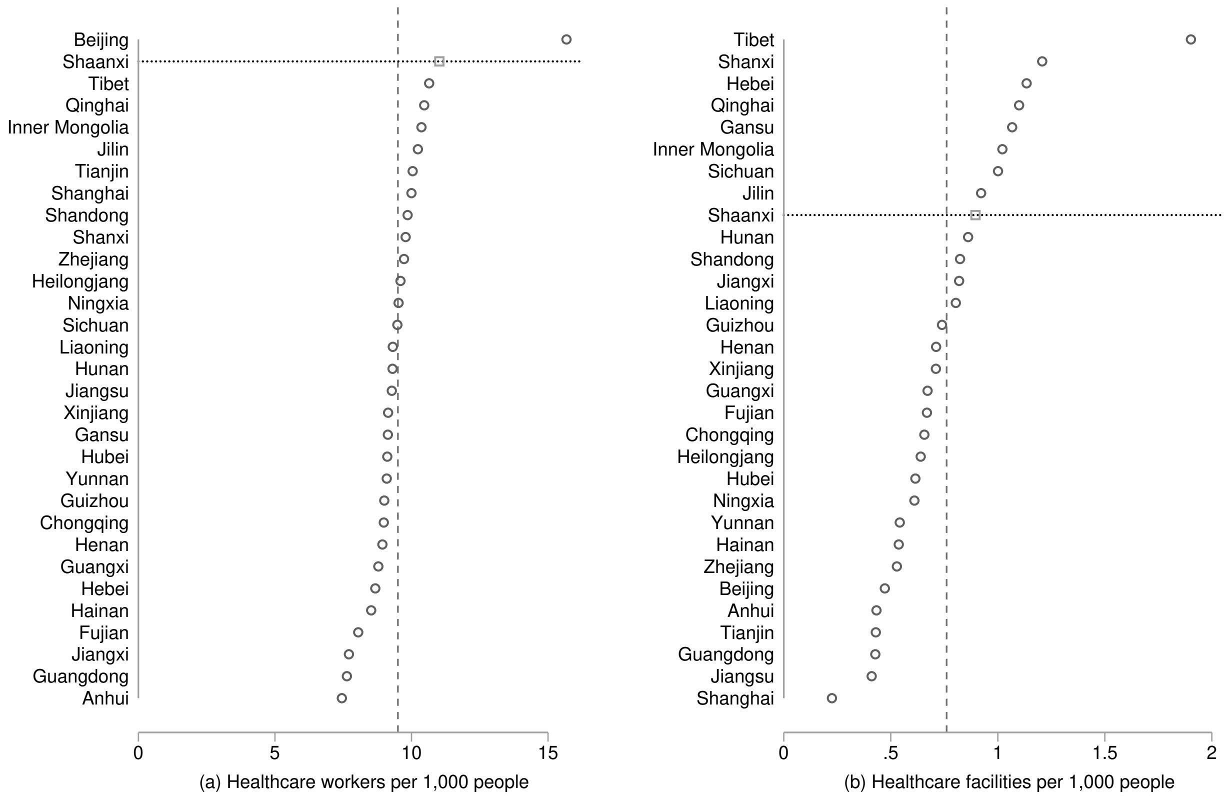

Note: Statistics of healthcare workers and healthcare facilities are obtained from the China medical and health statistic yearbook in 2020. Vertical lines indicates levels of national average

Figure S2. Number of fever clinics, healthcare workers and healthcare facilities in Shaanxi, China in 2020

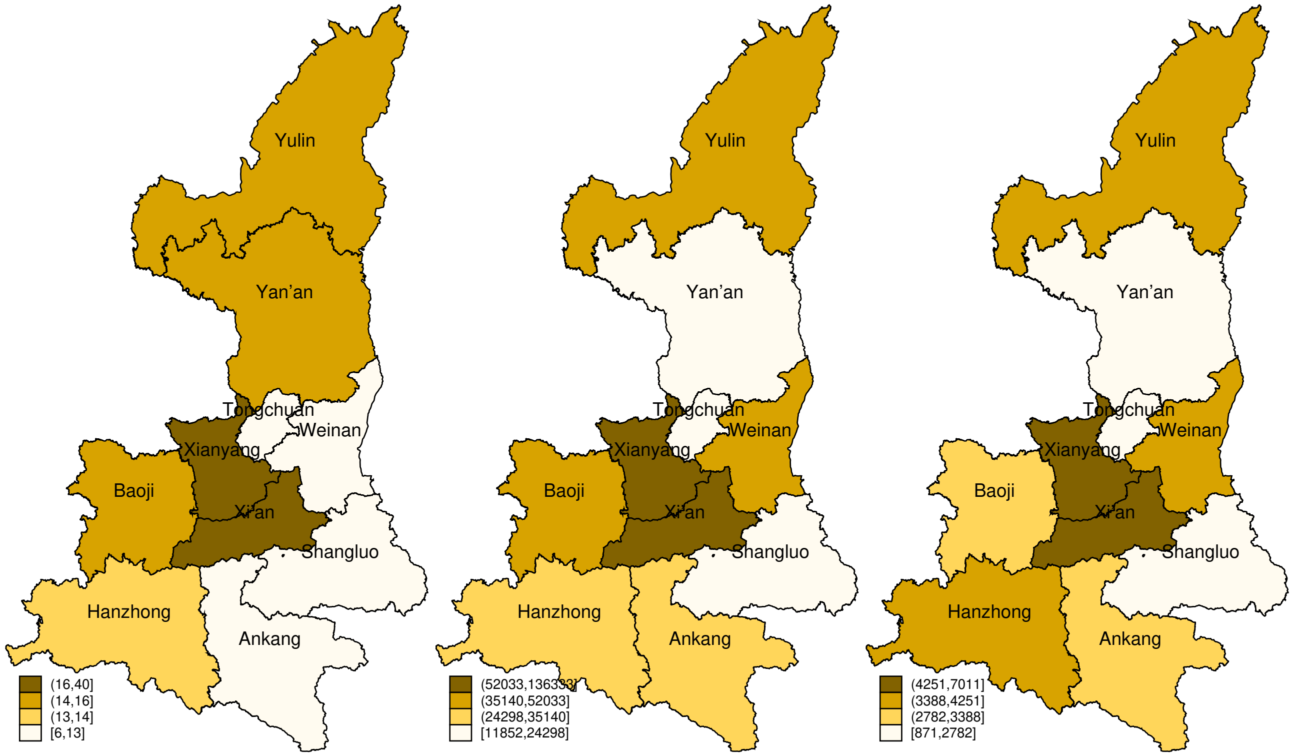

(a) Number of fever clinics (b) Number of healthcare workers (c) Number of healthcare facilities

Note: Statistics of fever clinics are extracted from published report by Shaanxi Health Commission and statistics of healthcare workers and healthcare facilities are obtained from the Shaanxi statistic yearbook in 2020.

Figure S3. Fever clinics per million, healthcare workers per thousand and healthcare facilities per thousand in Shaanxi, China in 2020

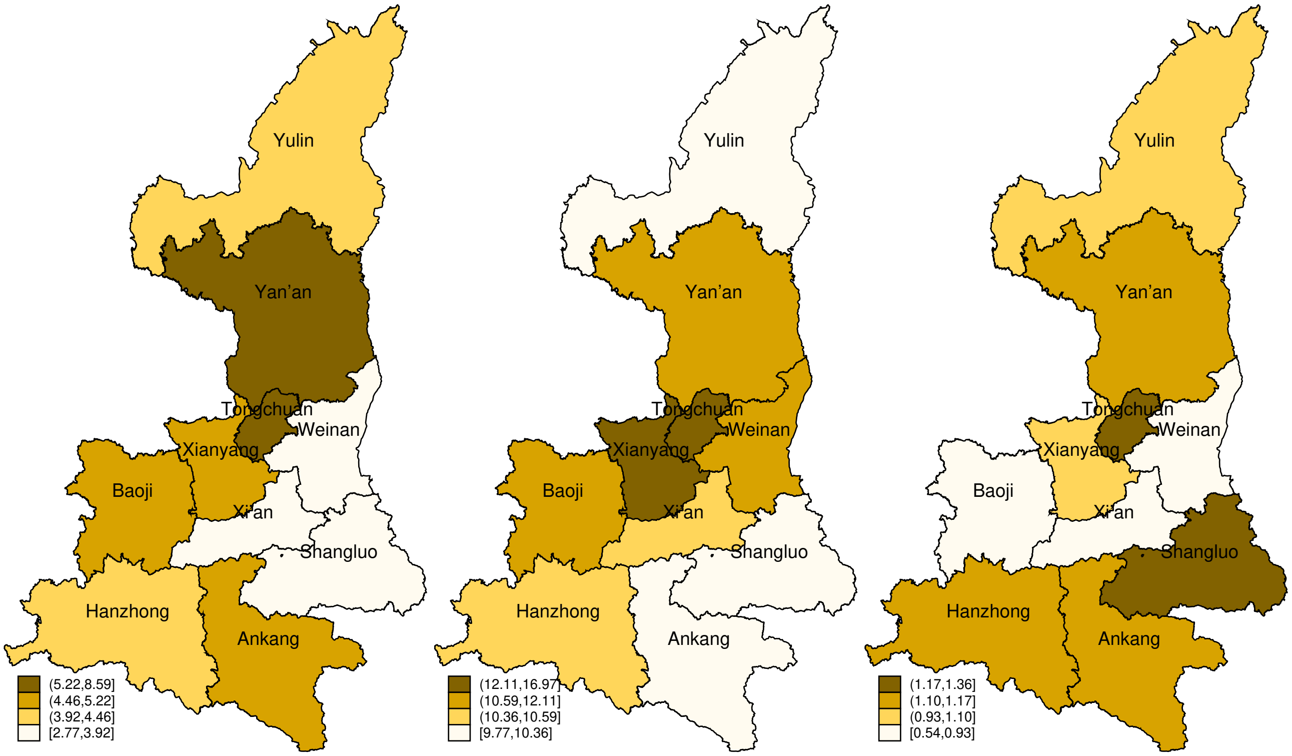

(a) Fever clinics per million (b) Healthcare workers per thousand (c) Healthcare facilities per thousand

Note: Statistics of fever clinics are extracted from published report by Shaanxi Health Commission and statistics of healthcare workers and healthcare facilities are obtained from the Shaanxi statistic yearbook in 2020.

References

1. The State Council of P.R.C. Notice on further strengthening responsibilities regarding COVID-19 prevention (in Chinese) (2020).
2. Xinhua News Agency. Fighting COVID-19: China in Action (2020).
3. WHO & Aylward, Bruce (WHO); Liang, W. P. Report of the WHO-China Joint Mission on Coronavirus Disease 2019 (COVID-19). Tech. Rep. (2020).
4. Zhang, Y., Zhao, Q. & Hu, B. Community-based prevention and control of COVID-19: Experience from China. *Am. J. Infect. Control.* DOI: 10.1016/j.ajic.2020.03.012 (2020).
5. Xinhua News Agency. China National Health Insurance Bureau: protection from high medical costs (in Chinese) (2020).
